# New criteria for estimating numbers of CD34-positive cells in a graft needed for posttransplant bone marrow recovery

**DOI:** 10.1038/s41375-024-02424-2

**Published:** 2024-09-25

**Authors:** Yahui Feng, Saibing Qi, Yu Hu, Wen Yan, Yanping Ji, Mingyang Wang, Xiaowen Gong, Qiujin Shen, Wei Zhang, Huilan Liu, Xianjing Zhang, Mengyun Chen, Erling Chen, Xiaolin Zhai, Yi He, Donglin Yang, Aiming Pang, Mingzhe Han, Robert Peter Gale, Zimin Sun, Erlie Jiang, Junren Chen

**Affiliations:** 1 State Key Laboratory of Experimental Hematology, National Clinical Research Center for Blood Diseases, Haihe Laboratory of Cell Ecosystem, Institute of Hematology & Blood Diseases Hospital, Chinese Academy of Medical Sciences & Peking Union Medical College, Tianjin, China; 2 Tianjin Institutes of Health Science, Tianjin, China; 3 Department of Hematology, Affiliated Hospital of Jiangsu University, Zhenjiang, China; 4 Department of Hematology, The First Affiliated Hospital of University of Science and Technology of China, Hefei, China; 5 Blood and Cell Therapy Institute, Division of Life Sciences and Medicine, Anhui Provincial Key Laboratory of Blood Research and Applications, University of Science and Technology of China, Hefei, China; 6 Centre for Haematology, Department of Immunology and Inflammation, Imperial College of Science, Technology and Medicine, London, UK

**Keywords:** Haematopoietic stem cells, Haematological diseases

## To the Editor:

In a previous, widely-read article in *LEUKEMIA* we discussed why, in umbilical cord blood (UCB) transplants, numbers of CD34-positive cells in a graft are best estimated by *blood volume* (BV) rather than by *body weight* (BW) [1]. We also showed, within the dose range we interrogated, there is no *threshold dose* for posttransplant bone marrow recovery using granulocyte recovery as a surrogate. These observations have important clinical implications which should change current clinical practice guidelines and expert consensus recommendations [2–5]. Following our publication, several experts quite reasonably asked whether these conclusions apply to conventional blood cell grafts. The answer, which is yes, follows.

To address this question we analyzed data from 746 consecutive subjects receiving blood cell transplants from HLA-matched siblings (Supplement Fig. 1; Supplementary Table 1). 635 were from the Institute of Hematology, Chinese Academy of Medical Sciences (Tianjin, China) 1 December, 2013–1 June, 2021 and 111 from the First Affiliated Hospital of University of Science and Technology of China (Hefei, China) 1 January, 2015–31 December, 2020.

391 (52%) recipients were male. Median age was 39 years (InterQuartile Range [IQR], 30–47 years). Median body height and weight were 166 cm (IQR, 160–172 cm) and 62 kg (IQR, 54–70 kg) with a median body mass index (BMI) of 23 (IQR, 20–25). Median estimated blood volume (calculated based on sex, age, body height and weight; https://skirt-calculator.shinyapps.io/CD34-positive_Cell_Dose_Calculator/ [1, 6]) was 4.0 L (IQR, 3.5–4.5 L). Correlation between body weight and estimated blood volume was 0.83 (95% Confidence Interval [CI], 0.81, 0.85).

Grafts were mobilized blood cells collected 4 days after starting granulocyte colony-stimulating factor (G-CSF). Median absolute total number of infused CD34-positive cells was 170 × 10E+6 (IQR, 137–227 × 10E+6). Granulocyte recovery was defined as reaching a granulocyte concentration > 0.5 × 10E+9/L that was sustained for ≥ 3 consecutive days whilst not receiving G-CSF. Median interval to granulocyte recovery was 13 days (IQR, 12–14 days). 5 subjects dying at a median of 14 days posttransplant (range, 7–16 days) before granulocyte recovery were right-censored and included in our analyses (Supplementary Table 2).^1^

**Fig. 1:**
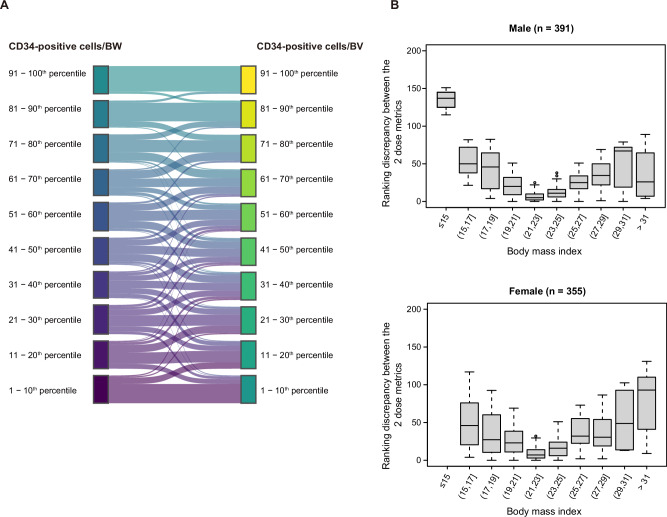
Comparison between CD34-positive cells/BW and CD34-positive cells/BV. **A** Correspondence between rankings (percentiles) of the two dose metrics. **B** The 2 dose metrics’ ranking discrepancy *versus* body mass index (BMI). Boxes indicate the 25th-, 50th- and 75th-percentile values. Whiskers indicate value ranges. Extreme values are separately represented by dots.

We compared 2 metrics for CD34-positive cell dose: CD34-positive cells/BW and CD34-positive cells/BV. Median CD34-positive cells/BW was 2.7 × 10E+6/kg (range, 0.7–19.8 × 10E+6/kg; IQR, 2.3–3.4 × 10E+6/kg) whilst median CD34-positive cells/BV, 4.2 × 10E+7/L (range, 0.9–27.3 × 10E+7/L; IQR, 3.5–5.5 × 10E+7/L). 92% of high CD34-positive cells/BW values (> 90th percentile) corresponded to high CD34-positive cells/BV values (> 90th percentile) whilst 69% of low CD34-positive cells/BW values (< 10th percentile) corresponded to low CD34-positive cells/BV values (< 10th percentile). In contrast, for values between the 10th and 90th percentiles correspondence between the 2 metrics was poor (40% matching; Fig. 1A). Rank discrepancy between CD34-positive cells/BW and CD34-positive cells/BV often occurred when the BMI was extreme. In both sexes, there was a U-shaped relationship between rank discrepancy and BMI (Fig. 1B).

Using Cox regression and assuming a linear relationship between CD34-positive cell dose and granulocyte recovery we estimated that for each 10E+6/kg increment in CD34-positive cells/BW the hazard for granulocyte recovery increased by 18% (*P* < 0.001). For each 10E+7/L increment in CD34-positive cells/BV the hazard increased by 12% (*P* < 0.001). These data indicate both metrics correlated significantly with speed of granulocyte recovery.

**Fig. 2:**
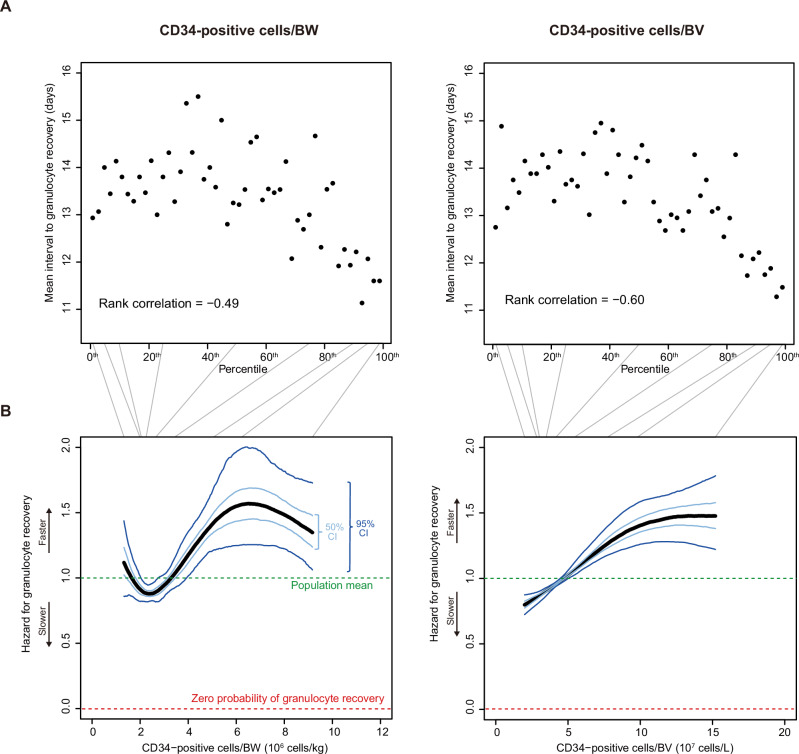
Relationship between CD34-positive cell dose and speed of granulocyte recovery. **A** Ranking (percentile) of CD34-positive cell dose *versus* mean interval to granulocyte recovery. Each dot represents an interval spanning 2 percentage points along the horizontal axis. **B** Non-linear hazard functions of the 2 dose metrics for granulocyte recovery. ‘CI’ stands for ‘confidence interval’. Gray lines indicate mappings between percentiles and dose values.

There was a clear trend mean interval to granulocyte recovery decreased as dose metric (CD34-positive cells/BW or CD34-positive cells/BV) increased (Fig. 2A). However, the rank correlation between mean interval to granulocyte recovery and CD34-positive cells/BW was –0.49 *versus* –0.60 with CD34-positive cells/BV (*P* = 0.03; boot-strapping test).

Removing linearity assumption we used Bayesian restricted cubic splines to estimate the relationship between CD34-positive cell dose and speed of granulocyte recovery [1, 7]. The hazard function of CD34-positive cells/BW for granulocyte recovery was not monotonously increasing and had up-and-down swings (Fig. 2B). Put otherwise, the lowest and highest CD34-positive cells/BW values did not correspond to the slowest and fastest rates of granulocyte recovery. In contrast, the hazard function of CD34-positive cells/BV was strictly monotonously increasing (Fig. 2B).

Interestingly, the hazard for granulocyte discovery stayed ≥ 0.8 for both dose metrics even when CD34-positive cell dose was much lower than the widely-accepted threshold of 4–5 × 10E+6/kg (Fig. 2B) [3, 5]. Consistent with concepts we previously discussed and within the dose range we interrogated there is no threshold dose of CD34-positive cells needed for granulocyte recovery, a surrogate for recovery of bone marrow function [1].

In sensitivity analyses focusing only on subjects with acute leukaemia or myelodysplastic neoplasms (*n* = 625) or only on those receiving intensive pretransplant conditioning (*n* = 650) our conclusions were unchanged (Supplement Figs. 2 and 3).

Elsewhere we discuss it is highly improbable numbers of CD34-positive cells would accurately correlate with numbers of haematopoietic stem cells [1]. Nonetheless, physicians may want an estimate of the speed of granulocyte recovery based on numbers of CD34-positive cells in a graft. Our data indicate numbers of CD34-positive cells/BV rather than CD34-positive cells/BW is the more accurate predictor of rate of granulocyte recovery, a surrogate of posttransplant bone marrow recovery and should replace CD34-positive cells/BW in updated clinical practice guidelines and expert consensus recommendations.

Equally important, our data indicate within the dose range we interrogated there is no threshold dose of CD34-positive cells/BW or /BV needed for posttransplant bone marrow recovery. This should make many more umbilical cord blood cell units usable for adults and prevent unnecessary repeat leukaphereses of blood cell transplant donors. This knowledge should result in revised clinical practice guidelines and expert consensus recommendations.

We acknowledge it is difficult or impossible to change ingrained physician practices. However, a Xianbei saying goes, *better titbits of jade than a slab of clay*.

## Supplementary information

Supplementary Materials

## Data Availability

Clinical data are available upon reasonable request to JC and consistent with the laws of China.

## Footnotes

1 2, 2 and 1 had aplastic anaemia, acute myeloid leukaemia (1 in 1st complete remission; 1 not in remission) and myelodysplastic neoplasm. 4 had CD34-positive cells/BW and CD34-positive cells/BV > the 25^th^-percentile values of the study cohort (> 2.3 × 10E+6/kg and > 3.5 × 10E+7/L). 4 had infection pretransplant and died of septic shock posttransplant. 1 died of cardiac failure starting pretransplant.

## Code availability

We described the computational algorithm in detail previously in the Supplemental Material of ref. [1].
