# Supplementary material for: New criteria for estimating numbers of CD34-positive cells in a graft needed for posttransplant bone marrow recovery
Source: Leukemia. 2024 Sep 25;38(12):2735–8. doi: 10.1038/s41375-024-02424-2 (PMC11588648; doi:10.1038/s41375-024-02424-2)
Supplement: Supplementary file 1 — Supplementary Materials [file 41375_2024_2424_MOESM1_ESM.docx]

New Criteria for Estimating Numbers of CD34-Positive Cells in a Graft Needed for Posttransplant Bone Marrow Recovery

Yahui Feng^1,2+^, Saibing Qi^1,2+^, Yu Hu^1,2+^, Wen Yan^1,2+^, Yanping Ji^3^, Mingyang Wang^1,2^, Xiaowen Gong^1,2^, Qiujin Shen^1,2^, Wei Zhang^1,2^, Huilan Liu^4,5^, Xianjing Zhang^1,2^, Mengyun Chen^1,2^, Erling Chen^4^, Xiaolin Zhai^1,2^, Yi He^1,2^, Donglin Yang^1,2^, Aiming Pang^1,2^, Mingzhe Han^1,2^, Robert Peter Gale^6^, Zimin Sun^4,5*^, Erlie Jiang^1,2*^, Junren Chen^1,2*^

^1^ State Key Laboratory of Experimental Hematology, National Clinical Research Center for Blood Diseases, Haihe Laboratory of Cell Ecosystem, Institute of Hematology & Blood Diseases Hospital, Chinese Academy of Medical Sciences & Peking Union Medical College, Tianjin, China

^2^ Tianjin Institutes of Health Science, Tianjin, China

^3^ Department of Hematology, Affiliated Hospital of Jiangsu University, Zhenjiang, China

^4^ Department of Hematology, The First Affiliated Hospital of University of Science and Technology of China, Hefei, China

^5^ Blood and Cell Therapy Institute, Division of Life Sciences and Medicine, Anhui Provincial Key Laboratory of Blood Research and Applications, University of Science and Technology of China, Hefei, China

^6^ Centre for Haematology, Department of Immunology and Inflammation, Imperial College of Science, Technology and Medicine, London, UK

^+^ Co-first authors

* Co-senior authors

**Supplement Table 1. Subject co-variates (n = 746).**

| **Year at transplant, n (%)** |  |
| --- | --- |
| 2013 | 5 (1) |
| 2014 | 71 (10) |
| 2015 | 95 (13) |
| 2016 | 106 (14) |
| 2017 | 102 (14) |
| 2018 | 80 (11) |
| 2019 | 126 (17) |
| 2020 | 120 (16) |
| 2021 | 41 (5) |
| **Age, years, median (IQR)** | 39 (30 – 47) |
| **Male, n (%)** | 391 (52) |
| **Height cm, median (IQR)** | 166 (160 – 172) |
| **Weight kg, median (IQR)** | 62 (54 – 70) |
| **BMI, kg/m^2^, median (IQR)** | 23 (20 – 25) |
| **Primary disease, n (%)** |  |
| Acute leukaemia | 475 (64) |
| Myelodysplastic syndromes | 150 (20) |
| Aplastic anaemia | 88 (12) |
| Others | 33 (4) |
| **Pretransplant conditioning, n (%)** |  |
| MAC | 650 (87) |
| RIC | 96 (13) |
| **ABO, n (%)** |  |
| Match | 461 (62) |
| Major mis-match | 128 (17) |
| Minor mis-match | 103 (14) |
| Bi-directional mis-match | 54 (7) |
| **CD34-positive cells/BW, 10E+6/kg, median (IQR)** | 2.7 (2.3 – 3.4) |
| **CD34-positive cells/BV, 10E+7/L, median (IQR)** | 4.2 (3.5 – 5.5) |
| **Posttransplant immune suppression, n (%)** |  |
| CSA-based | 452 (61) |
| TAC-based | 294 (39) |
| **Survival, % (95-percent CI)** |  |
| 180 days | 92 (90 – 94) |
| 1 year | 84 (82 – 87) |
| 3 years | 76 (72 – 79) |

­­­­

Abbreviations: BMI, body mass index; BV, blood volume; BW, body weight; CI, confidence interval; CSA, cyclosporine A; IQR, InterQuartile Range; MAC, myeloablative conditioning; RIC, reduced-intensity conditioning; TAC, tacrolimus.

**Supplement Table 2. Subjects dying before granulocyte recovery**

| **UPN** | 1 | 2 | 3 | 4 | 5 |
| --- | --- | --- | --- | --- | --- |
| **Age, years** | 27 | 53 | 52 | 44 | 52 |
| **Sex** | Male | Male | Male | Female | Female |
| **ECOG score** | NA | NA | 4 | 1 | 3 |
| **Diagnosis** | Aplastic anaemia | AML | Aplastic anaemia | AML | MDN |
| **Disease state** | - | NR | - | CR1 | - |
| **Donor** | HLA-matched sibling | HLA-matched sibling | HLA-matched sibling | HLA-matched sibling | HLA-matched sibling |
| **ABO-match** | Match | Match | Major  mis-match | Match | Bi-directional mis-match |
| **Donor sex** | Female | Female | Male | Male | Female |
| **Pretransplant**  **conditioning** | RIC | MAC | RIC | MAC | MAC |
| **CD34-positive cells/BW, 10E+6/kg** | 2.5 | 2.3 | 5.2 | 2.4 | 2.0 |
| **CD34-positive cells/BV, 10E+7/L** | 3.5 | 3.6 | 7.0 | 4.6 | 2.8 |
| **Cause of death** | Infection | Infection | Infection | Cardiac failure | Infection |
| **Death (d)** | +14 | +7 | +15 | +13 | +16 |

­­­­

Abbreviations: AML, acute myeloid leukaemia; BV, blood volume; BW, body weight; CR1, 1st complete remission; ECOG, Eastern Cooperative Oncology Group; MAC, myeloablative conditioning; MDN, myelodysplastic neoplasm; NA, not available; NR, not remission; RIC, reduced-intensity conditioning; UPN, unique patient number.

**Supplement Figure 1. CONSORT flow diagram.**

**
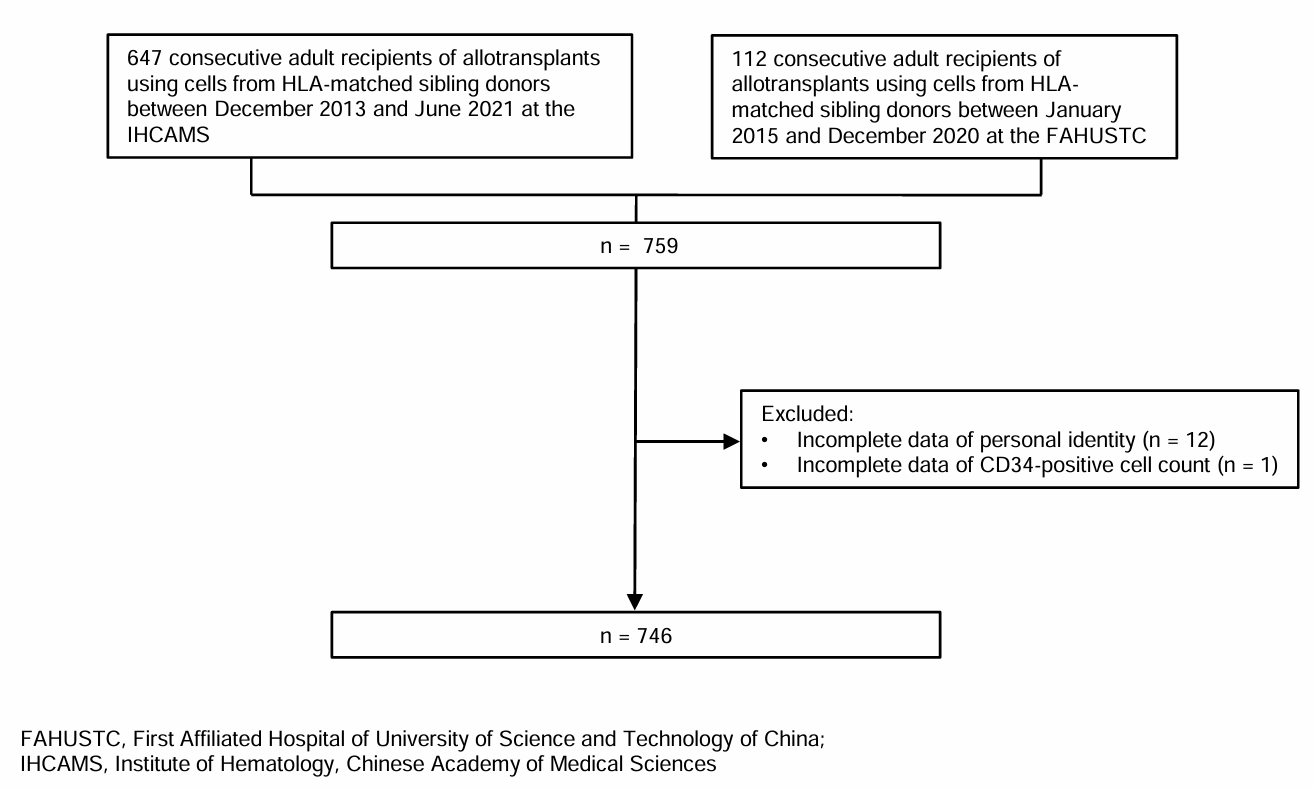
**

**Supplement Figure 2. Relationship between CD34-positive cell dose and speed of granulocyte recovery in the sub-cohort of recipients with acute leukaemia or myelodysplastic syndromes (n = 625).**


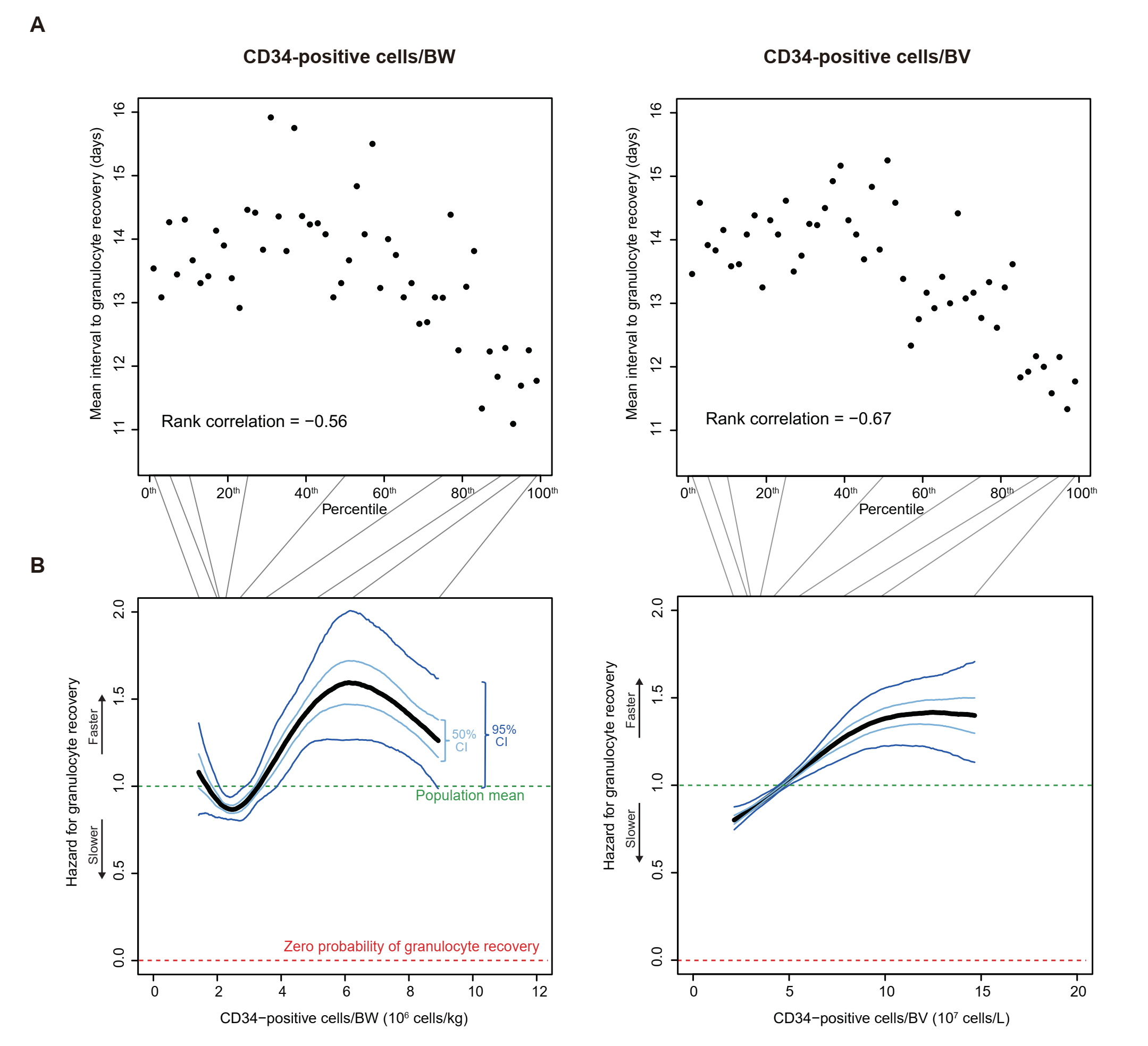


Difference between the 2 rank correlations in Panel A: *P* = 0.03 (boot-strapping test).

**Supplement Figure 3. Relationship between CD34-positive cell dose and speed of granulocyte recovery in the sub-cohort with intensive (‘myelo-ablative’) pretransplant conditioning (n = 650).**


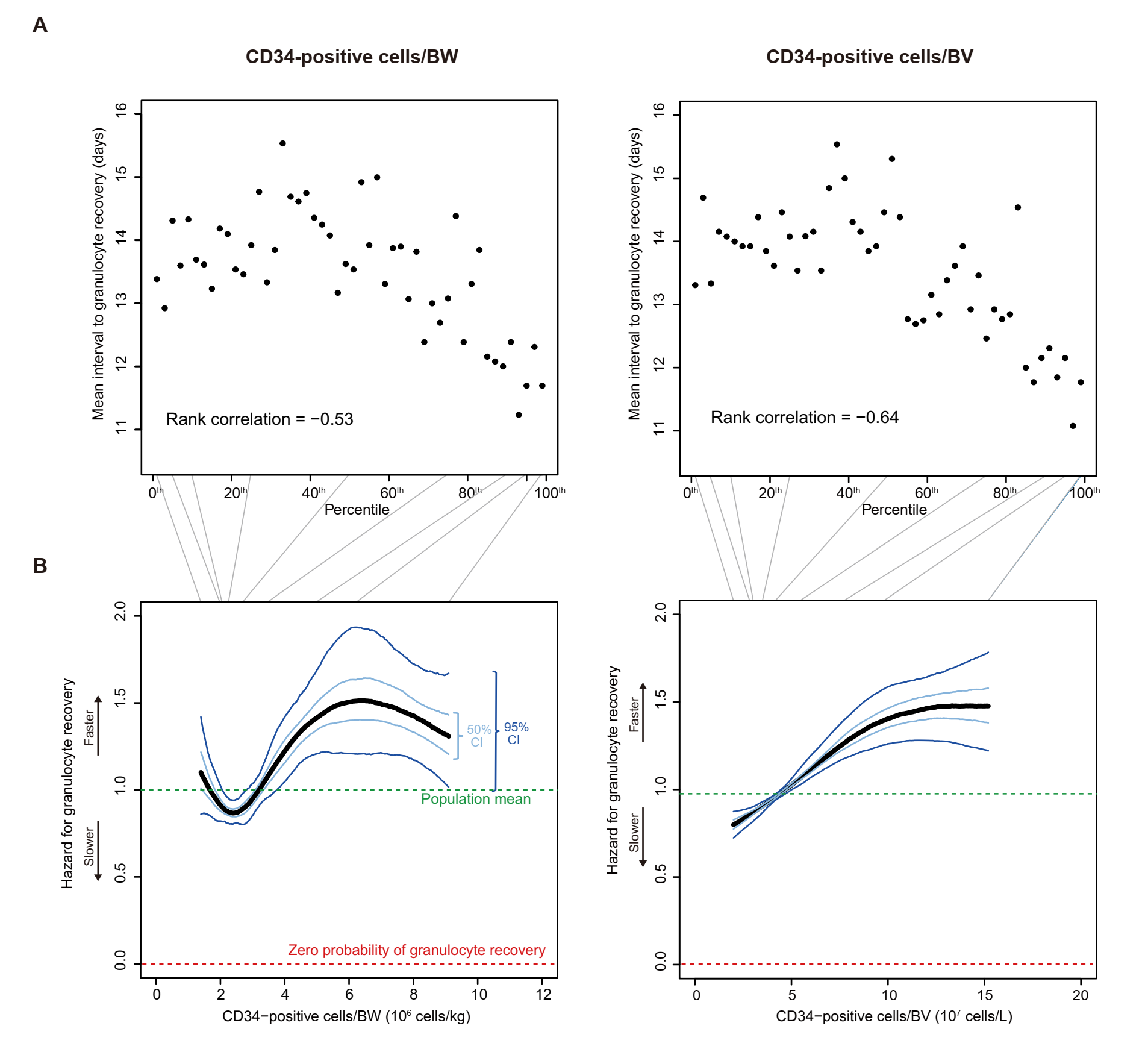


Difference between the 2 rank correlations in Panel A: *P* = 0.03 (boot-strapping test).
